# Antibacterial Susceptibility Patterns and Cross-Resistance of Acinetobacter, Isolated from Hospitalized Patients, Southern Iran

**Published:** 2011-11-01

**Authors:** S Japoni, S Farshad, A Abdi Ali, A Japoni

**Affiliations:** 1 Department of Biology, Alzahra University, Tehran, Iran; 2 Professor Alborzi Clinical Microbiology Research Center, Nemazee hospital, Shiraz University of Medical Sciences, Shiraz, Iran

**Keywords:** Acinetobacter, Antibiotic resistance, Cross resistance, Iran

## Abstract

**Background:**

Acinetobacter is a multi-drug resistant and nosocomial pathogen. The aim of this study was to determine antibacterial susceptibility patterns and cross-resistance of Acinetobacter species.

**Methods:**

This study was conducted in Nemazee Hospital, Shiraz, Iran from October 2007 to September 2008. Species identification was carried out by API E20. Minimum inhibitory concentration and cross-resistance of the isolated strains to 12 antibiotics were determined by E-test method.

**Results:**

Eighty eight isolates of Acinetobacter were collected from patients’ samples. Acinetobacter baumannii was isolated most frequently (79; 89.8%). Colistin, imipenem and meropenem were found to be the three most effective antibiotics with 97.7%, 77.3% and 72.7% activity against the isolates, respectively. Multi-drug resistance was revealed among 2 to 11 antibiotics and high cross-resistance was also noticed.

**Conclusion:**

To alleviate the situation, strict control measures and appropriate effective antibiotic therapy should be adopted to reduce hospital costs and related mortality.

## Introduction

Acinetobacter is a gram-negative, strictly aerobic, non-motile, non-fermentative, oxidase negative, catalase positive and citrate positive bacterium. Most strains can grow in a simple mineral medium containing single carbon and energy source.[1] Acinetobacter baumannii is more frequent in clinical samples, while A. lwoffii and A. haemolyticus were also isolated in environmental settings. Infections due to A. baumannii are frequently found in the intensive care units (ICUs), where they are implicated as the cause of ventilator-associated pneumonia (VAP), urinary tract infections, and bacteremia. In clinical practices, Acinetobacter infections are influenced by various risk factors including the use of medical devices such as endotracheal tubes, intravascular and urinary catheters, the exposure to broad-spectrum antibiotics and the ICU wards where the patients are admitted and the infection rate is high.[2] Resistance rates to fluoroquinolones, aminoglycosides, cephalosporins, and penicillins are high in several regions. Although carbapenem remains mainstay of the therapy for suspected Acinetobacter infections, resistance to this antimicrobial class has been increasingly reported. Thus, therapeutic options can become markedly limited.[3][4]

Major hospital outbreaks, related to multi-drug resistant (MDR) Acinetobacter spp. have been recently described in several countries, making surveillance of antimicrobial susceptibility an important public health task.[5] This study was conducted to assess the prevalence of different Acinetobacter species and their Correspondence frequencies in samples. Furthermore, susceptibility patterns and cross-resistance of Acinetobacter to different antibiotics were determined.

## Materials and Methods

This study was a cross-sectional one in which bacteria identification was carried out by standard biochemical tests; API E20 (BioMerieux, Marcy I, Etoile, France), in Nemazee Hospital, Shiraz, Iran from October 2007 to September 2008. Briefly, the isolates which were mostly obtained from blood, urine wound and sputum, were sub-cultured on blood agar and MacConkey agar to ensure viability and purity. The isolates were stored at –20ºC in nutrient broth containing 50% v/v glycerol. Minimum inhibitory concentration (MIC) of the isolates to 12 antibiotics including ciprofloxacin, colistin, ceftazidime, imipenem, ampicillin/sulbactam, meropenem, gentamicin, norfloxacin, amikacin, cefepime, tobramycin and cefoperazon/sulbactom were determined by E-test method and interpreted as recommended by the manufacturer's instruction. Cross-resistance of antibiotic resistant isolates to different antibiotics was also evaluated.

The data were analyzed statistically by SPSS software version 15 (SPSS, Chicago, IL, USA). Cross-resistance was obtained by cross tabulation of the resistant samples within SPSS software.

## Results

Eighty eight isolates of Acinetobacter were obtained. Acinetobacter spp. was isolated predominantly from men (70%), as compared to women (30%). The samples consist of; 35 (39.8%) blood, 15 (17%) wound, 15 (17%) sputum, 13 (14.8 %) urine and 10 (11.4%) samples of CSF, eyes and joints. Acinetobacter bau¬mannii strains were isolated most frequently (79; 89.8%), followed by A. lwoffii (8; 9.1%) and A. hae¬molyticus (1; 1.1%).

Based on the susceptibility of the isolated Acinetobacter to the 12 antibiotics, colistin, imipenem and meropenem proved to be the three most effective antibiotics with 97.7%, 77.3% and 72.7% activities against the isolates, respectively. These data were collected in Table 1. Comparison of MICs for the two main isolated species revealed that A. baumannii spp. was more resistant, compared to A. lwoffii (Table 1). Acinetobacter baumannii was resistant between 2 to 11 antibiotics of which resistance rates of 6, 7 and 8 antibiotics were observed predominantly (Table 2).To more precisely determine the resistance patterns of A. baumannii to the tested antibiotics, cross-resistance of the isolates was calculated and presented in Table 3. High cross-resistance was noticed to the majority of the tested antibiotics.

**Table 1:** In vitro susceptibility patterns of 88 Acinetobacter spp. to 12 antibiotics and comparison of susceptibility values for A. baumannii and A. lwoffii^a^.

**Antibiotics**	**Total *Acinetobacter spp. *No. 88**		***A. baumannii* No. 79**		***A. lwoffii *No. 8**	
	**R (%)**	**S (%)**	**R (%)**	**S (%)**	**R (%)**	**S (%)**
PM	71 (80.7)	17 (19.3)	70 (88.6)	9 (11.4)	0 (0)	8 (100)
TZ	72 (81.8)	16 (18.2)	69 (87.3)	10 (12.6)	2 (25)	6 (75)
GM	70 (79.5)	18 (20.5)	69 (87.3)	10 (12.6)	0 (0)	8 (100)
NX	67 (76.1)	21 (23.9)	67 (84.8)	12 (15.2)	0 (0)	8 (100)
AK	66 (75)	22 (25)	65 (82.3)	14 (17.7)	1 (12.5)	7 (87.5)
CI	65 (73.9)	23 (26.1)	65 (82.3)	14 (17.7)	0 (0)	8 (100)
TM	32 (36.4)	56 (63.6)	31 (39.2)	48 (60.7)	0 (0)	8 (100)
CPS	29 (33)	59 (67)	29 (36.7)	50 (63.3)	0 (0)	8 (100)
AB	34 (38.6)	54 (61.4)	22 (27.8)	57 (72.1)	1 (12.5)	7 (87.5)
MP	24 (27.2)	64 (72.7)	22 (27.8)	57 (72.1)	0 (0)	8 (100)
IP	20 (22.7)	68 (77.3)	18 (22.8)	61 (77.2)	1 (12.5)	7 (87.5)
CO	2 (2.3)	86 (97.7)	1 (1.3)	78 (98.7)	0 (0)	8 (100)

^a^ Abbreviations: TZ, ceftazidime; GM, gentamicin; NX, norfloxacin ; AK, amikacin; CI, ciprofloxacin; TM, tobramycin; CPS, cefoperazon/sulbactom AB, ampicillin/sulbactam; MP, meropenem; IP, imipenem; CO, colistin; R, resistant; S, sensitive.

**Table 2:** Frequencies and patterns of multi-resistant isolates of Acinetobacter to the tested antibiotics.^a^

**Resistant antibiotics**	**Antibiotic resistance pattern **	**No. **	**Total**
0	Sensitive	12	12
1	TZ	1	1
2	TZ-AK	1	4
	AB-PM	1	
	AK-TM	1	
	GM-TM	1	
3	AB-IP-MP	1	1
5	NX-TZ-GM-TM-PM	1	2
	TZ-GM-AK-TM-PM	1	
6	NX-TZ-GM-CI-AK-PM	13	17
	NX-TZ-GM-CI-AB-PM	2	
	NX-TZ-GM-AK-TM-PM	1	
	NX-TZ-CI-AK-MP-PM	1	
7	NX-TZ-GM-CI-AK-CPS-PM	2	17
	NX-TZ-GM-CI-AK-TM-PM	14	
	NX-TZ-GM-CI-AK-PM-CO	1	
8	NX-TZ-GM-AK-IP-TM-MP-PM	1	18
	NX-TZ-GM-CI-AB-AK-CPS-PM	11	
	NX-TZ-GM-CI-AK-TM-MP-PM	2	
	NX-TZ-GM-CI-AB-AK-TM-PM	1	
	TZ-GM-AB-IP-TM-MP-PM-CO	1	
	TZ-GM-CI-AK-IP-TM-MP-PM	1	
	NX-TZ-GM-CI-AB-AK-TM-MP	1	
9	NX-TZ-GM-CI-AB-IP-CPS-MP-PM	2	2
10	NX-TZ-GM-CI-AB-AK-IP-CPS-MP-PM	6	6
11	NX-TZ-GM-CI-AB-AK-IP-TM-CPS-MP-PM	8	8
Total	26 (patterns)	88	88

^a^ Abbreviations: CI, ciprofloxacin; CO, colistin; TZ, ceftazidime: AB, ampicillin/sulbactam; IP, imipenem; MP, meropenem; GM, gentamicin; NX, norfloxacin; AK, amikacin: PM, cefepime; TM, tobramycin; CPS, cefoperazon/sulbactom

**Table 3:** Cross-resistance of Acinetobacter to the tested antibiotics.^a^

		**Number of isolates and percent (value in parenthesis) resistant to**											
	**No.**	**NX **	**TZ **	**GM **	**CI **	**AB **	**AK **	**IP **	**TM **	**CPS **	**MP **	**PM **	**CO **
NX	67		67 (100)	66 (98.5)	64 (95.5)	31 (46.3)	62 (92.5)	17 (25.4)	27 (40.3)	29 (43.3)	21 (31.3)	67 (100)	1 (1.5)
TZ	72	67 (93.1)		69 (95.8)	65 (90.3)	32 (44.4)	65 (90.3)	19 (26.4)	30 (41.7)	29 (40.3)	23 (31.9)	70 (97.2)	2 (2.8)
GM	70	66 (94.3)	69 (98.6)		64 (91.4)	32 (45.7)	63 (90)	19 (27.1)	31 (44.3)	29 (41.4)	22 (31.4)	69 (98.6)	2 (2.9)
CI	65	64 (98.5)	65 (100)	64 (98.5)		31 (47.7)	61 (93.8)	17 (26.2)	25 (38.5)	29 (44.6)	21 (32.3)	65 (100)	1 (1.5)
AB	34	31 (91.2)	32 (94.2)	32 (94.2)	31 (91.2)		27 (79.4)	18 (52.9)	10 (29.4)	27 (79.4)	19 (55.9)	33 (97.1)	1 (2.9)
AK	66	62 (93.9)	65 (98.5)	63 (95.4)	61 (92.4)	27 (40.9)		16 (24.2)	29 (43.90)	27 (40.9)	20 (30.3)	64 (96.9)	1 (1.5)
IP	20	17 (85)	19 (95)	19 (95)	17 (85)	18 (90)	16 (84.2)		10 (50)	16 (84.2)	20 (100)	19 (95)	1 (5)
TM	32	27 (84.4)	30 (93.7)	31 (96.9)	25 78.1)	10 (31.2)	29 (90.6)	10 (31.2)		7 (21.9)	13 (40.6)	30 (93.7)	1 (3.1)
CPS	29	29 (100)	29 (100)	29 (100)	29 (100)	27 (93.1)	27 (93.1)	16 (55.2)	7 (24.1)		16 (55.2)	29 (100)	0 (0)
MP	24	21 (87.5)	23 (95.8)	22 (91.7)	21 (87.5)	19 (79.2)	20 (83.3)	20 (83.3)	13 (54.2)	16 (66.7)		23 (95.8)	1 (4.2)
PM	71	67 (94.4)	70 (98.6)	69 (97.2)	65 (91.5)	33 (46.5)	64 (90.1)	19 (26.8)	30 (42.2)	29 (40.8)	23 (32.4)		2 (2.8)
CO	2	1 (50)	2 (100)	2 (100)	1 (50)	1 (50)	1 (50)	1 (50)	1 (50)	0 (0)	1 (50)	2 (100)	

^a^ Abbreviations: NX, norfloxacin; TZ, ceftazidime ; GM, gentamicin ;CI, ciprofloxacin; AB, ampicillin/sulbactam ;AK, amikacin; IP, imipenem; TM, tobramycin ; CPS, cefoperazon/sulbactom ; MP, meropenem;PM, cefepime; CO, colistin.

## Discussion

Various biochemical and molecular methods to identify Acinetobacter have been applied. According to these methods, these bacteria are categorized into several genomic groups, but only a few have received genomic names.[6][7][8] In this study, 89.8% of the isolates were A. baumannii and 10.2% were non-A. baumanni (9.1% A. lwoffii and 1.1% A. haemolyticus). Based on a study by Feizabadi et al. in Tehran, 84.4% of the samples were A. baumannii and 15.6% were non-A. Baumannii.[9] Similar to the current results, Seifert et al. reported that 72.9% of the clinical specimens were A. baumannii and the rest were non-A. Baumannii.[10] Most samples in the present study were isolated from the blood (39.8%) and the rest were isolated from wound, sputum, urine and other sites. In Feizabadi et al. report, blood samples were more frequent (37.7 %). Predomination of Acinetobacter from blood samples may indicate the role of bloodstream in disseminating the infection.[11]

In the present study, A. baumannii with high resistance to different classes of antibiotics including fluoroquinolones, aminoglycosides and cephalosporins were detected. In agreement with this survey, multi-drug resistant Acinetobacter was isolated from the hospitalized patients worldwide.[12][13][14][15][16][17] Nevertheless, A. lwoffii expressed low resistance to the most of tested antibiotics. Low resistance of A. lwoffii could be due to the absence of efficient antibiotic resistance capturing system (integron) or as a result of its low dissemination in hospital environment. It has been proven that A. baumannii has infected prolonged hospitalized patients.[18][19] Acinetobacter spp. was highly sensitive to colistin (97.7%) and moderately sensitive to imipenem (77.3%) and meropenem (72.7%). Despite the high sensitivity of Acinetobacter to colistin, its use should be restricted to life-threatening conditions because of serious neurological and renal side effects.[20][21]

The high antibiotic resistance observed in the present study, could be due to the extensive clinical administration of antibiotics. Acquisition of antibiotic resistance in Acinetobacter is a result of transferable resistance elements such as plasmid, integron and transposon.[22] Of the mentioned factors, integron has received more attention in developing antibiotic resistance due to high efficient capturing system.[22][23][24][25] To alleviate the situation, periodical determination of the regional antibiotic susceptibility patterns of Acinetobacter is recommended. Furthermore, strict control measures and appropriate effective antibiotic therapy should be adopted. The majority of the isolated Acinetobacter exhibited cross-resistance to the tested antibiotics (Table 3), and consequently limited the application of effective antibiotics, in cases empiric therapy needs to be considered or alternative therapy has indication. Determination of the source of infection in the hospitals using Pulse Field Gel Electrophoresis (PFGE) and constant training of the medical staff to control the infection are also recommended and could be helpful.

## Conclusion

In conclusion, due to the high possibility of transmission of the antibiotic resistance through a variety of transmissible elements such as plasmid, transposon and specially integron, reduction in the prevalence of multi-drug resistant bacteria in clinics and hospitals is mandatory. By taking comprehensive control measures and making rational prescription of appropriate antibiotics, the situation could be improved accordingly to an acceptable level.
